# EMG-Driven Forward-Dynamic Estimation of Muscle Force and Joint Moment about Multiple Degrees of Freedom in the Human Lower Extremity

**DOI:** 10.1371/journal.pone.0052618

**Published:** 2012-12-26

**Authors:** Massimo Sartori, Monica Reggiani, Dario Farina, David G. Lloyd

**Affiliations:** 1 Department of Neurorehabilitation Engineering, Bernstein Focus Neurotechnology, University Medical Center Göttingen, Georg-August University, Göttingen, Germany; 2 Institute of Biomedical Engineering, National Research Council, Padova, Italy; 3 Department of Management and Engineering, University of Padova, Vicenza, Italy; 4 Centre for Musculoskeletal Research, Griffith Health Institute, Griffith University, Gold Coast, Queensland, Australia; The University of Western Ontario, Canada

## Abstract

This work examined if currently available electromyography (EMG) driven models, that are calibrated to satisfy joint moments about one single degree of freedom (DOF), could provide the same musculotendon unit (MTU) force solution, when driven by the same input data, but calibrated about a different DOF. We then developed a novel and comprehensive EMG-driven model of the human lower extremity that used EMG signals from 16 muscle groups to drive 34 MTUs and satisfy the resulting joint moments simultaneously produced about four DOFs during different motor tasks. This also led to the development of a calibration procedure that allowed identifying a set of subject-specific parameters that ensured physiological behavior for the 34 MTUs. Results showed that currently available single-DOF models did not provide the same unique MTU force solution for the same input data. On the other hand, the MTU force solution predicted by our proposed multi-DOF model satisfied joint moments about multiple DOFs without loss of accuracy compared to single-DOF models corresponding to each of the four DOFs. The predicted MTU force solution was (1) a function of experimentally measured EMGs, (2) the result of physiological MTU excitation, (3) reflected different MTU contraction strategies associated to different motor tasks, (4) coordinated a greater number of MTUs with respect to currently available single-DOF models, and (5) was not specific to an individual DOF dynamics. Therefore, our proposed methodology has the potential of producing a more dynamically consistent and generalizable MTU force solution than was possible using single-DOF EMG-driven models. This will help better address the important scientific questions previously approached using single-DOF EMG-driven modeling. Furthermore, it might have applications in the development of human-machine interfaces for assistive devices.

## Introduction

Human movement is the result of the actuation of joints in upper and lower extremities. Joints are actuated by the coordinated excitation of musculotendon units (MTUs). The multiple MTUs spanning a joint reflect the redundancy of the human neuromuscular system in which a prescribed joint moment and motion can be the result of different MTU excitation strategies. Understanding how this redundancy is solved in humans and how MTUs develop force during movement is one of the biggest challenges in biomechanics. This has been previously studied using optimization-driven methodologies in which MTUs are assumed to contribute to the experimentally measured joint moments according to a chosen criterion that is presumed to be generalizable across subjects and motor tasks [1], [2], [3]. However, it has been shown that in humans the neuromuscular redundancy is solved by means of the neural drive to MTUs, or MTU excitation. In this scenario, MTUs are recruited independently of the final joint moment and position, but rather based on the motor task to be performed [4], [5], [6], and on the personal history of training and pathology [7], [8].

Surface electromyography (EMG) indirectly reflects the dynamics of an individual's neural drive and can be easily recorded during human movement [9]. For this reason experimentally recorded EMG signals have been used to directly drive simulations of upper and lower extremity musculoskeletal models as an alternative solution to optimization-driven methods [10], [11], [12], [13], [14].

EMG-driven musculoskeletal modeling (EMG-driven modeling) is a forward dynamics approach. In this, the EMG data experimentally recorded from the major muscle groups are used to drive multiple MTUs within a subject-specific physiologically accurate model of the human musculoskeletal system. In this scenario, the recorded EMG data directly determine the patterns of MTU excitation and the resulting MTU force and moment. This approach has the advantage, over optimization-driven methods, of solving the neuromuscular redundancy problem based on an individual's estimate of the neural drive without having to make assumptions on how MTUs share the load about a joint.

EMG-driven modeling requires an off-line calibration to determine a number of model parameters that vary non-linearly across subjects because of anatomical and physiological differences [11]. During the calibration step, a nominal set of parameters is initially used in the model to predict the MTU force and the resulting joint moment as a function of EMG signals during a set of calibration trials. The initial parameter set is repeatedly refined until the mismatch between predicted and experimental joint moments is minimized. Once an optimal, subject-specific, parameter set if found, the calibrated EMG-driven model is validated on a novel set of trials that was not used during calibration [10], [11], [12], [15]. During the validation step the calibrated EMG-driven model behaves as an open-loop predictive system. That is, it predicts MTU forces and the resulting joint moments as a function of measured EMG signals and joint kinematics without the need to track experimental joint moments.

The currently available state of the art EMG-driven modeling methodologies employ calibration to create a robust model that only accounts for one selected degree of freedom (DOF) of the human limbs and for the associated MTUs spanning the specific DOF. In this, the activity of the selected MTUs is constrained to satisfy the joint moment or motion for the only selected DOF. Such models (i.e. single-DOF models) have been designed for the elbow flexion-extension [16], knee flexion-extension [11], [13], [17], and ankle plantar-dorsi flexion [18], [19]. When run in open-loop (i.e. after calibration), single-DOF models have been shown to well predict joint moments about the DOF for which the model was calibrated. However, even though single-DOF models account for the neuromuscular redundancy based on experimental EMG data, it has never been examined whether they can be applied to predict the MTU dynamics and the resulting joint moment with respect to a different DOF than that used for calibration. In other words, is the force, generated by the same MTU and driven by the same input data during the same movement, predicted differently if different single-DOF models are used? In this scenario it is worth accounting for the fact that errors may be introduced when calibrating an EMG-driven model with respect to a DOF (i.e. hip flexion-extension) and using it to predict the action of MTUs and the resulting joint moment with respect to a different DOF (i.e. hip adduction-abduction). Single-DOF calibrations could therefore result in models that would predict substantially different force estimates for the same MTU and for the same data set of EMG and joint kinematics. This could represent a major limitation of currently available single-DOF models and would pose the question of how to combine together solutions from different single-DOF models if the predictions across the MTUs that are shared by different single-DOF models do not match.

Based on the above-mentioned considerations, this study had three aims. The first aim was to verify if the currently available EMG-driven models, which were calibrated about a single DOF, could provide the same unique MTU force solution when calibrated with respect to different single DOFs. Second, because it was found that currently available single-DOF calibrations did not result in the same calibrated EMG-driven model, we examined whether the development of a more comprehensive model that accounted for multiple DOFs and for a larger set of MTUs could address limitations in single-DOF EMG-driven modeling. To-this-end, we developed a multi-DOF EMG-driven musculoskeletal model (multi-DOF model) of the human lower extremity. Our proposed model used EMG signals recorded from 16 muscle groups to drive 34 MTUs and produce a single force solution that satisfied joint moments generated around four DOFs including hip adduction-abduction (HipAA), hip flexion-extension (HipFE), knee flexion-extension (KneeFE), and ankle plantar-dorsi flexion (AnkleFE). This led to the development of a more comprehensive calibration procedure that allowed identifying a set of subject-specific parameters that ensured physiological behavior for the large MTU set. Third, we examined whether the multi-DOF calibrated model could predict joint moments, of the included DOFs, with comparable accuracy with respect to each individual model calibrated about a single-DOF. We then examined and compared the force solution produced by the multi-DOF and single-DOF models for both MTUs acting about one or more joints and DOFs.

Single-DOF EMG-driven modeling has been consistently applied to answer questions in many scientific areas, ranging from motor control to injury development, from biomechanics to rehabilitation robotics [16], [18], [19], [20], [21], [22], [23], [24]. However, for these questions to be addressed properly, the development of multi-DOF EMG-driven modeling is crucial. This will allow attaining confidence that the predicted MTU force solution is generalizable across DOFs, i.e. it satisfies joint moments with respect to multiple DOFs simultaneously. Furthermore, it will allow predicting and analyzing the dynamics of a larger set of MTUs than it was possible with current single-DOF models. This will provide a more reliable means of MTU force estimation from experimental EMG data resulting in a deeper understanding of the neuromuscular dynamics during the human movement. Finally, it may open-up to the development of robust neuromuscular human-machine interfaces for the simultaneous and proportional control of multiple DOFs in wearable assistive devices such as powered orthoses and prostheses.

## Methods

### Data collection and analysis procedures

One healthy male subject (age: 28 years, height: 183 cm, mass: 67 kg) volunteered for this investigation and gave his informed, written consent. The project was approved by the Human Research Ethics committee at the University of Western Australia.

The motion data acquired from the subject were static anatomical poses and dynamic gait trials. During all trials, the three-dimensional location of retro-reflective markers placed on the subject's body was recorded (250 Hz) using a 12-camera motion capture system (Vicon, Oxford, UK). During the dynamic trials, ground reaction forces (GRFs) and EMG data were collected (2000 Hz) synchronously with marker trajectories using an in-ground force plate (AMTI, Watertown, USA), and bipolar electrodes with a telemetered EMG system (Noraxon, Scottsdale, USA) respectively. Both GRFs and marker trajectories were low-pass filtered with a fourth-order Butterworth filter. Cut-off frequencies (between 2 and 8 Hz) were determined by a trial-specific residual analysis [25]. EMGs were processed by band-pass filtering (10–450 Hz), then full-wave rectifying and low-pass filtering (6 Hz). The resulting linear envelopes were normalized with respect to the peak processed EMG values obtained from the entire set of recorded trials.

From the dynamic trials collected, two distinct datasets were created; one for the calibration of the single-DOF and multi-DOF models and the other one for the validation. The calibration dataset included two repeated trials of four motor tasks including walking (WK) (1.3±0.25 m/s), running (RN) (2.5±0.5 m/s), sidestepping (SS) (1.9±0.35 m/s), and crossover (CO) (1.8±0.15 m/s) cutting maneuvers. A different dataset was used to validate the calibrated EMG-driven models. This included ten repeated novel trials for each of the four considered motor tasks (WK, RN, SS, and CO). The novel trials used for the model validation were performed at the same speeds as those used for the model calibration. However, none of the trials in the validation dataset were included in the calibration dataset, i.e. there was no intersection of data between the two datasets. The four motor tasks were chosen because 1) they allowed producing substantially high moments (i.e. always greater than 50 Nm) about the four considered DOFs (HipAA, HipFE, KneeFE, and AnkleFE), and 2) because they reflected different MTU recruitment strategies and contraction dynamics. This allowed investigating whether our proposed multi-DOF model could predict joint moments simultaneously produced about the four considered DOFs while accounting for different MTU operation strategies.

Using the software OpenSim [26], a generic model of the human musculoskeletal geometry [2] was scaled to match the individual subject's size and body proportions. During this process, virtual markers were created and placed on the musculoskeletal geometry model based on the position of the experimental markers recorded from the static standing poses. In this process, the anthropomorphic properties of the anatomical segments and MTUs were linearly scaled based on the relative distances between experimental markers and their corresponding virtual markers. The adjusted segment and MTU properties included: anatomical segment length, width, depth, center of mass location, and mass moment of inertia, as well as MTU insertion, origin, and MTU-to-bone wrapping points. In addition, certain MTU parameters (e.g. tendon slack length, optimal fiber length, and maximum isometric force) were adjusted to the individual using the procedure outlined in the upcoming *Multi-DOF EMG-driven Model* section [14].

After the anthropomorphic scaling, the OpenSim Inverse Kinematics (IK) algorithm [26] solved for joint angles that minimized the least-squared error between experimental and virtual markers. The joint moments that needed to track the IK-generated angles were obtained using Inverse Dynamics (ID) and Residual Reduction Analysis (RRA) [26]. The joint moments produced by this pathway were called “the experimental” moments. The alternate pathway to estimate the joint moments was by the EMG-driven model.

### Multi-DOF EMG-driven Model

**Figure 1:**
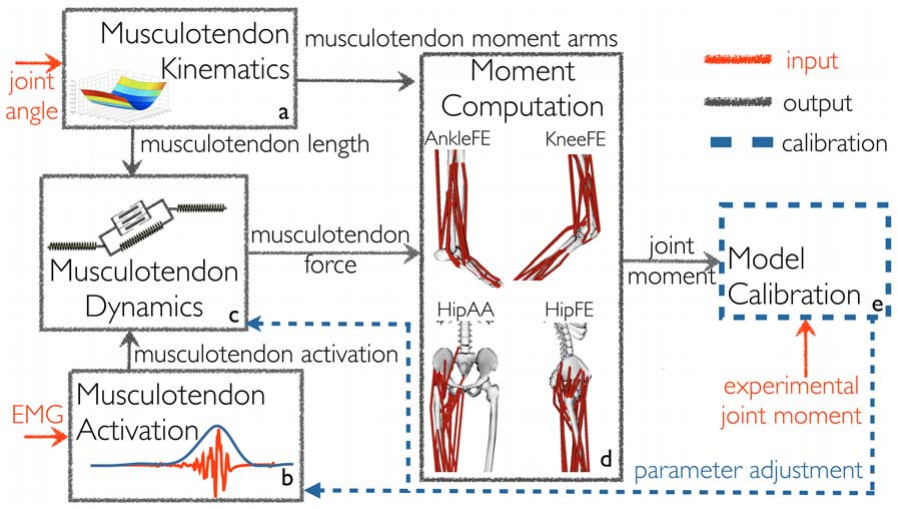
The schematic structure of the multi-DOF EMG-driven model. It comprises of five components: a) Musculotendon Kinematics, b) Musculotendon Activation, c) Musculotendon Dynamics, d) Moment Computation, and e) Model Calibration Process. The multi-DOF EMG-driven model is initially calibrated using the Model Calibration component. After calibration the EMG-driven model is operated in open-loop. Musculotendon units force and the resulting moments are determined as a function of EMG signals and three-dimensional joint angles, without tracking experimental joint moments. Joint moments are predicted with respect to four degrees of freedom (DOFs): hip adduction-abduction (HipAA), hip flexion-extension (HipFE), knee flexion-extension (KneeFE), and ankle plantar-dorsi flexion (AnkleFE).

The multi-DOF EMG-driven model was developed from the previously published KneeFE single-DOF (KneeFE-DOF) model [11] and comprises five main components (Figure 1): Musculotendon Kinematics, Musculotendon Activation, Musculotendon Dynamics, Moment Computation, and Model Calibration.

The Musculotendon Kinematics component (Figure 1a) used MTU-specific multidimensional spline functions to produce instantaneous estimates of MTU length 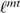, and three-dimensional moment arms 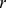 as a function of joint angles [27].

**Figure 2:**
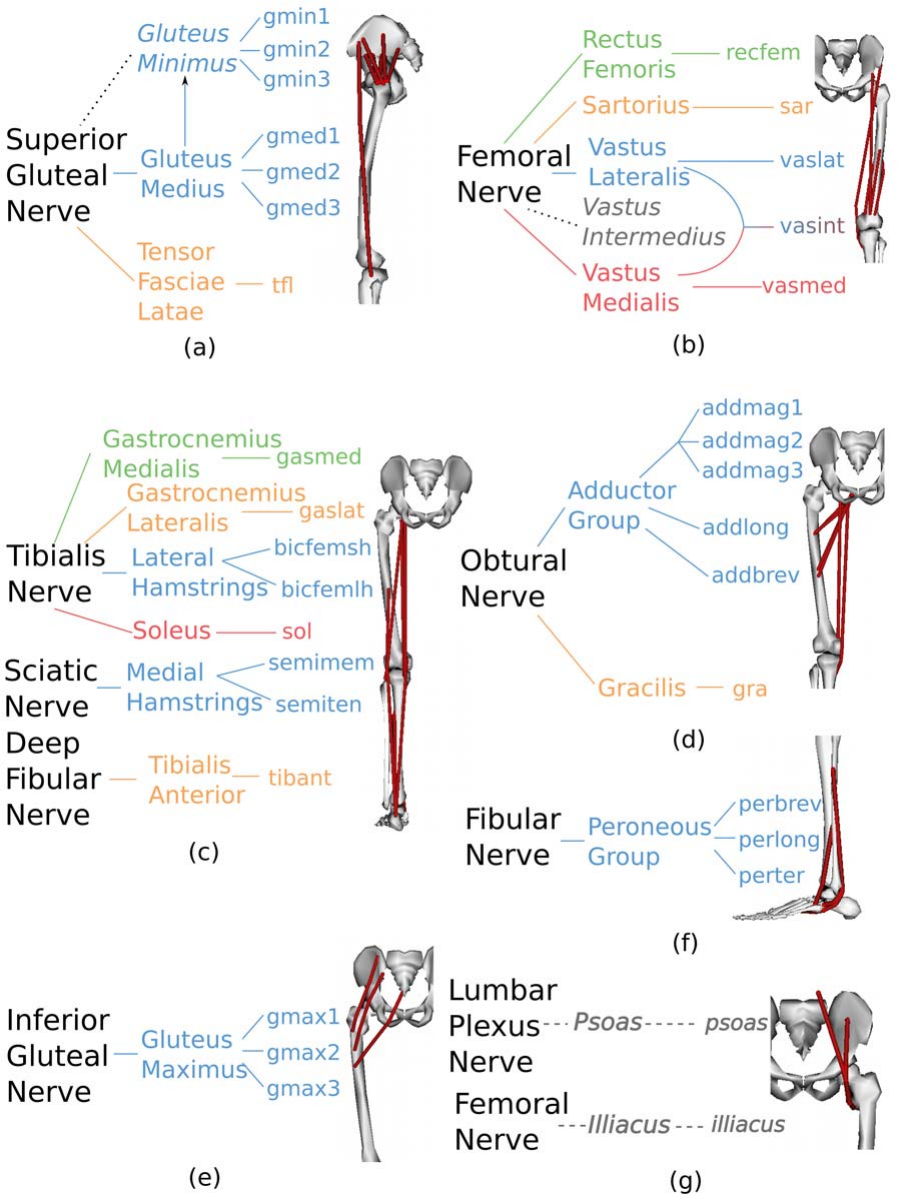
Allocation of experimental EMG signals to individual musculotendon units. The first level of each *tree* represents innervation zones in the human lower extremity. The second level represents the 18 muscle groups that are innervated from the corresponding innervation zone. At the second level, *italic-style* written names, connected by a dotted line to the first level, represent muscle groups for which experimental electromyography (EMG) signals could not be recorded (a, b, and g). The remaining 16 groups at the second level represent the muscles from which EMG signals were experimentally recorded. The third level represents the associated musculotendon units (MTUs) within each muscle group. All MTU names were abbreviated from the associated muscle group names in the second level with exception of the biceps femoris long and short head (bicfemlh, bicfemsh), semimembranosus (semimem), semitendinosus (semiten), adductor magnus, longus, and brevis (addmag, addlong, addbrev), and peroneus longus, brevis and tertius (perlong, perbrev, pertert). The gluteus minimus, medius, maximus, and the adductor magnus are modeled by three individual MTU compartments. Within each tree, branches have different colors referring to EMG signals recorded separately.

The Musculotendon Activation component (Figure 1b) allocated the EMG linear envelopes *e(t)* experimentally measured from 16 muscle groups to 32 MTUs in the model (Figure 2). In this allocation, two muscle groups that shared the same innervation and contributed to the same mechanical action were assumed to have the same EMG pattern [28]. This was then used to drive the corresponding MTUs within each muscle group. According to this convention the gluteus medius EMGs also drove the gluteus minimus MTUs. The lateral hamstring EMGs drove both the biceps femoris short head and long head MTUs. The medial hamstring EMGs drove both the semimembranosus and the semitendinosus MTUs. The adductor group EMGs drove the adductor magnus, longus and brevis MTUs. The peroneous group EMGs drove the peroneus longus, brevis and tertius MTUs. The vastus intermedius EMG activity was derived as the mean between the vastus lateralis and vastus medialis EMGs [10], [11], [29]. EMG signals could not be recorded from the illiacus and psoas MTUs that were too deep for surface recordings. However, we modeled their passive elastic force contribution, as it was found to be substantially high during the validation trials: 131±9N across CO trials, 125±9N across SS trials, 246±4N across RN trials, and 143±7N across WK trials. The passive elastic force of the illiacus and psoas MTUs was quantified during the validation trials using the Musculotendon Dynamics component (Figure 1c) and by setting the MTU activation to zero. This allowed predicting the resistive force produced by these MTUs to compression and stretching as a function of joint angle. The remaining lower extremity muscles were not accounted for because they were too deep for EMG recording and had a small physiological cross-sectional area. The MTUs in the model with HipAA, HipFE, KneeFE, and AnkleFE moment arms accounted for the 91%, 87%, 95%, and 80% of the total physiological cross-sectional area respectively. The MTU-allocated 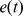 were then processed by a recursive filter to model the MTU twitch response to the EMG onset and were further adjusted to account for the non-linear EMG-to-force relationship [10], [11]. The resulting signal was called the MTU activation 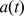.

In the Musculotendon Dynamics component (Figure 1c), each MTU had fibers modeled using generic force-velocity 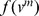, force-length passive 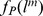, and active 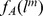 curves. These were normalized to maximum isometric muscle force (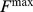), optimal fiber length, and maximum muscle contraction velocity [30]. The tendon dynamics was modeled using a non-linear force-strain function 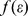
normalized to 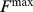 [30]. Using biomechanical parameters from [31], the MTU force 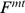 was calculated as a function of 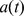, fiber length 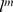, and fiber contraction velocity 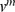:
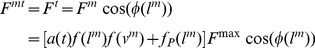
(1)where 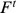 and 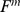 were the tendon and fiber force, and 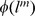 was the pennation angle that changed with instantaneous fiber length assuming the muscle belly had a constant thickness and volume [11]. At each time frame, 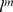
was determined from 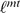 so to guarantee the equilibrium between 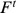 and 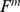
in Equation 1 [11]. Finally, the Moment Computation component (Figure 1d) estimated the joint moments 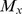 as the sum of the product of 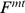 and 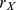, with X ∈ (HipAA, HipFE, KneeFE, AnkleFE).

The Model Calibration process (Figure 1e) determined the values for a set of parameters that vary non-linearly across subjects and cannot be determined experimentally or from the literature. The initial parameter values were taken from the literature [32] and were subsequently adjusted to the individual subject by varying them within predefined boundaries. This ensured MTUs always operated within their physiological range [11]. Parameters were adjusted using a simulated annealing algorithm [33] until the objective function 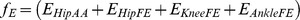 was minimized equally for each DOF. Each DOF error term 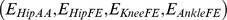 was the sum of the root mean square differences between the predicted and experimental joint moments calculated over the eight calibration trials. Calibration for the single-DOF models only adjusted the parameters for the MTUs associated to the single DOF *X* of interest so to minimize the associated single fitting error term 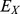 [11].

In the Musculotendon Activation component two global (i.e. it applies to all MTUs) activation filtering coefficients were constrained to vary between −1 and 1 to realize a stable positive solution and a critically damped impulsive response for the recursive filter [10], [11], [12]. One global shape factor parameter was also altered between −5 and 0 to account for the non-linear EMG-to-force relationship [11].

In the Musculotendon Dynamics component, muscle strength coefficients were adjusted and used to scale the MTU-specific 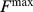 to maintain the relative strength across MTUs [11], [14]. Strength coefficients were varied between 0.5 to 2 and gathered MTUs in 11 groups according to their functional action including uniarticular hip flexors, uniarticular hip extensors, hip adductors, hip abductors, uniarticular knee flexors, uniarticular knee extensors, uniarticular ankle plantar flexors, uniarticular ankle dorsi flexors, biarticular quadriceps, biarticular hamstrings, and biarticular calf muscles.

MTU-specific tendon slack lengths 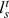, and optimal fiber lengths 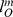 were also adjusted in the Model Calibration. However, prior to calibration, initial values for these parameters were found using the preferred scaling method (i.e. the seventh method) among the ones presented by Winby et al. [14]. This method adjusted the initial values of 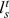 and 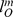, obtained from literature [8], [29], [31], [32], so that the muscle fiber and the tendon functional operating ranges were preserved between the generic and scaled musculoskeletal models [14]. However, this scaling assumed the subject being investigated had MTU properties of an average healthy individual. Therefore, these initial values were further adjusted in the Model Calibration to better reflect the actual subject's MTU intrinsic properties [10], [11], [13], [14], [16]. During this process, parameters were constrained to vary so that 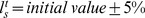 and 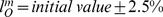.

### Validation Procedure

The validation comprised three tests to assess the single-DOF and multi-DOF models prediction ability and one to assess the computation time. In the three tests for prediction ability, the calibrated multi-DOF and single-DOF models predicted 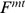 and 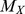 solely using experimental EMG and joint angle data from the stance phase during the 40 validation trials. Data from the same motor task were time-normalized using a cubic spline and averaged across trials, producing motor task-specific ensemble average curves for the predicted 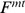, 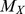, and for the matching experimental joint moments 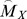.

The first test assessed whether single-DOF models produced different force estimates for the same MTU using the same input data and during the same movement. If single-DOF calibrated models did not produce a unique MTU force solution, and if the predictions across the MTUs that are shared by different single-DOF models did not match, then this would imply the impossibility of combining together solutions from different single-DOF models and predict joint moments about multiple DOFs simultaneously. For this purpose, the 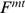 for a MTU spanning *D* DOFs (e.g. HipAA, HipFE and KneeFE for the rectus femoris), was predicted and averaged during the 40 validation trials, using the *D* associated single-DOF models. This produced *D* ensemble average 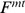 curves for each MTU. The *D* ensemble average 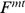 curves were then arranged into all possible 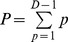 pairs. A pair-specific coefficient of the squared Pearson product moment correlation 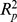 was then calculated as an index of shape similarity between the two single-DOF models solutions in the specific pair *p* being considered. The normalized root mean squared deviation (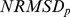) was also calculated for every pair *p* to reflect differences in magnitude:
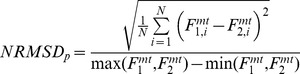
(2)where 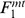 and 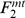
represented the forces predicted by two single-DOF models in the specific pair being considered. The pair-specific 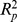 and 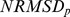 coefficients were then further averaged across pairs giving inter-item coefficients 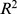 and *NRMSD* per MTU. In this analysis, uniarticular knee and ankle MTUs were not accounted for because they only had one associated single-DOF model.

The second test compared the joint moment prediction accuracy of the multi-DOF model to that of the four single-DOF models using the task-specific ensemble average curves. To-this-end, the mean absolute error (MAE) and its standard deviation (σ) were calculated:
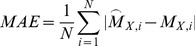
(3)where *N* refers to the number of points in the ensemble average curves. Furthermore, a measure of the percentage MAE (%MAE) was calculated by dividing Equation 3 with respect to the range of variation assumed by the experimental joint moment 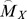, (i.e. 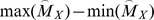).

The third test compared the multi-DOF model 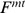 solutions to those obtained by the single-DOF models. The 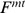 for a MTU spanning *D* DOFs was computed using the multi-DOF and the associated single-DOF models. Then, for each MTU, the 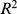 and *NRMSD* coefficients were calculated between the multi-DOF model 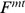 solution and each of the single-DOF models 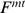 solutions respectively.

In the fourth test the multi-DOF model calibration and execution time were examined. Calibration time was calculated as the time needed to calibrate the multi-DOF model on the eight calibration trials. Execution time was calculated as the average time needed to compute one time point from all DOF joint moments 1000 times repeatedly. Tests were performed on an 8 GB RAM Intel i7 CPU.

## Results

**Figure 3:**
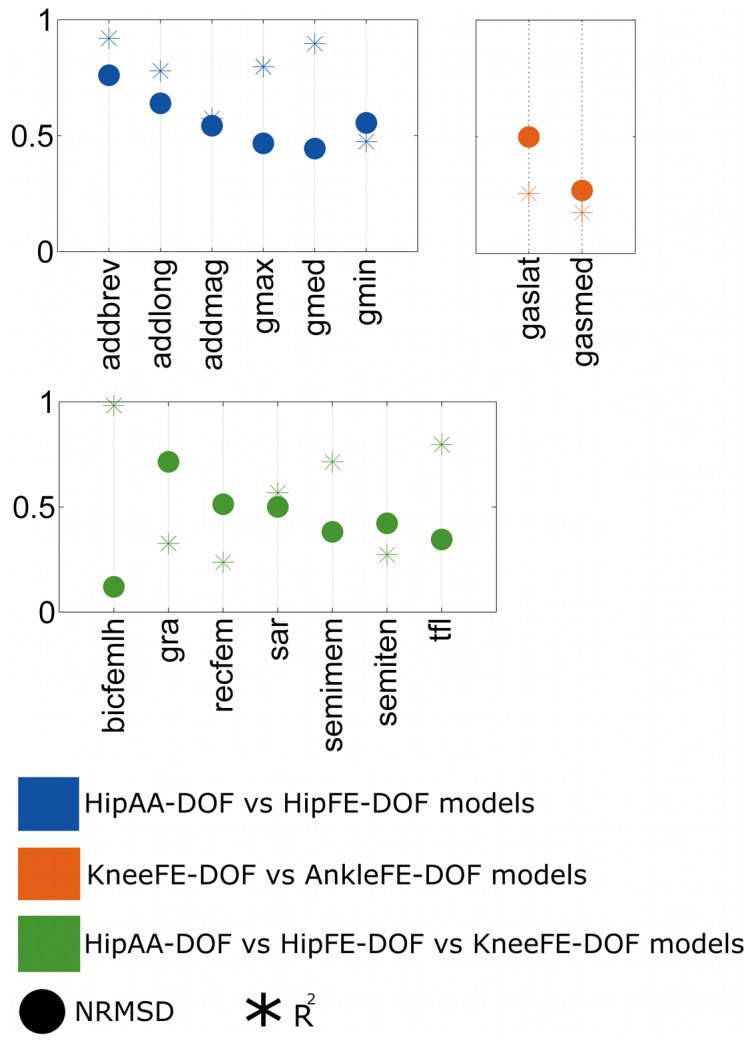
First test results: comparing MTU force estimates across single-DOF models. Square of the Pearson product moment correlation coefficient (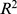) and normalized root mean squared deviation (NRMSD) between musculotendon unit (MTU) forces predicted by different single degree of freedom (DOF) models including: hip adduction-abduction (HipAA), hip flexion-extension (HipFE), knee flexion-extension (KneeFE), and ankle plantar-dorsi flexion (AnkleFE) single-DOF models. The MTU names abbreviations are defined in Figure 2. Values for the addmag, gmin, gmax, and gmed have been reported as the average between the individual values associated to the three units each muscle is composed of (Figs. 2a, d, and e).

The first test (Figure 3) revealed substantial differences, both in shape and magnitude, for 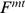 predicted by different single-DOF models. A comparison between the HipAA-DOF and the HipFE-DOF models showed 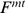 solutions with the weakest shape similarity for the adductor magnus 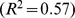 and gluteus minimus 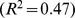. A stronger shape similarity was observed in the adductor longus 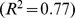 and gluteus maximus 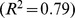, with values above 0.89 for the gluteus medialis and adductor brevis. However, in terms of magnitude, the HipAA-DOF and HipFE-DOF models 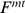 solutions were substantially different for all MTUs with *NRMSD* ranging between 0.45 and 0.77.

The 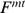 estimated using the HipAA-DOF, HipFE-DOF, and KneeFE-DOF models for the biarticular hip-knee MTUs had the lowest shape correlations for the gracilis 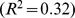, rectus femoris 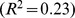, semitendinosus 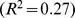, and sartorius 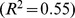 to which also corresponded substantially high magnitude differences (*NRMSD* = 0.72, 0.51, 0.43, and 0.51 respectively). The single-DOF solutions for the semimembranosus, tensor fasciae latae, and biceps femoris long head assumed good shape similarities (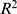 = 0.71, 0.79, and 0.98) and low magnitude differences (*NRMSD* = 0.38, 0.35, and 0.12).

The 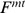 estimated using the KneeFE-DOF and AnkleFE-DOF models for the biarticular knee-ankle MTUs had poor shape correlations both for the gastrocnemius lateralis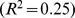 and medialis 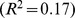 to which corresponded magnitude differences of *NRMSD* = 0.5 and *NRMSD* = 0.27 respectively.

**Figure 4:**
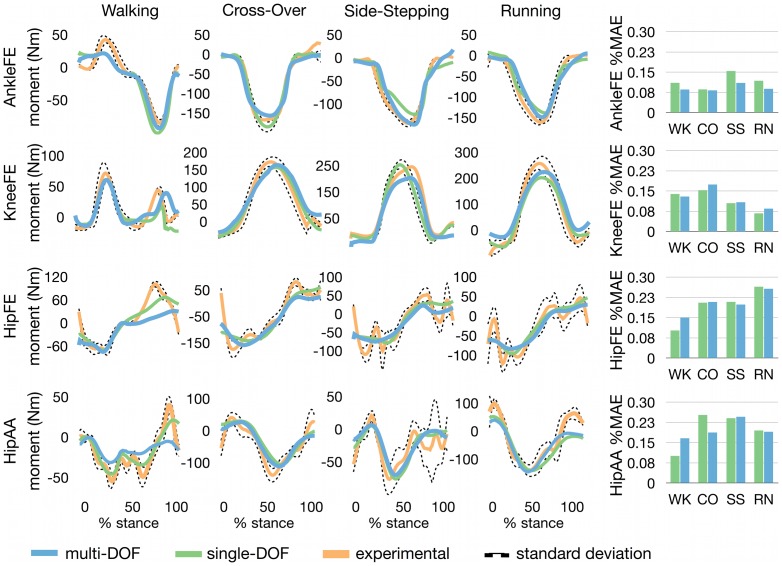
Second test results: comparing joint moment estimates between multi-DOF and single-DOF models. Ensemble average curves with associated standard deviation for the experimental joint moments about four degrees of freedom (DOF) including: hip adduction-abduction (HipAA), hip flexion-extension (HipFE), knee flexion-extension (KneeFE), and ankle plantar-dorsi flexion (AnkleFE). The reported data are from the stance phase with 0% being heel-strike and 100% toe-off events. The ensemble average curves are also reported for the matching joint moment predicted by the four corresponding single-DOF models and by the multi-DOF model. The percentage mean absolute error (%MAE) is reported in a histogram form and quantifies the percentage error between the experimental joint moments and those predicted by the multi-DOF model and by the four single-DOF models respectively. Ensemble average curves and %MAEs are shown for four motor tasks including: walking (WK), running (RN), side-stepping (SS), and cross-over (CO) cutting maneuvers.

In the second test (Figure 4), the multi-DOF model predicted joint moments simultaneously produced about four DOFs during the four considered motor tasks. Furthermore, the multi-DOF model predicted moments, at each included DOF, with comparable performance to the four single-DOF models. The %MAEs (and associated MAEs respectively) in joint moments estimation (histograms in Figure 4) for the four DOFs were (mean and SD across tasks) 0.12±0.03 (17.95±3.83 Nm) for the AnkleFE-DOF model and 0.09±0.01 (14.19±3.55 Nm) for the multi-DOF model, 0.12±0.04 (23.75±5.89 Nm) for the KneeFE-DOF model and 0.12±0.05 (26.22±4.88 Nm) for the multi-DOF model, 0.2±0.04 (26.4±7.82 Nm) for the HipFE-DOF model and 0.2±0.07 (27.99±8.02 Nm) for the multi-DOF model, 0.2±0.07 (27.39±11.38 Nm) for the HipAA-DOF model, and 0.2±0.03 (26.06±11.26 Nm) for the multi-DOF model.

**Figure 5:**
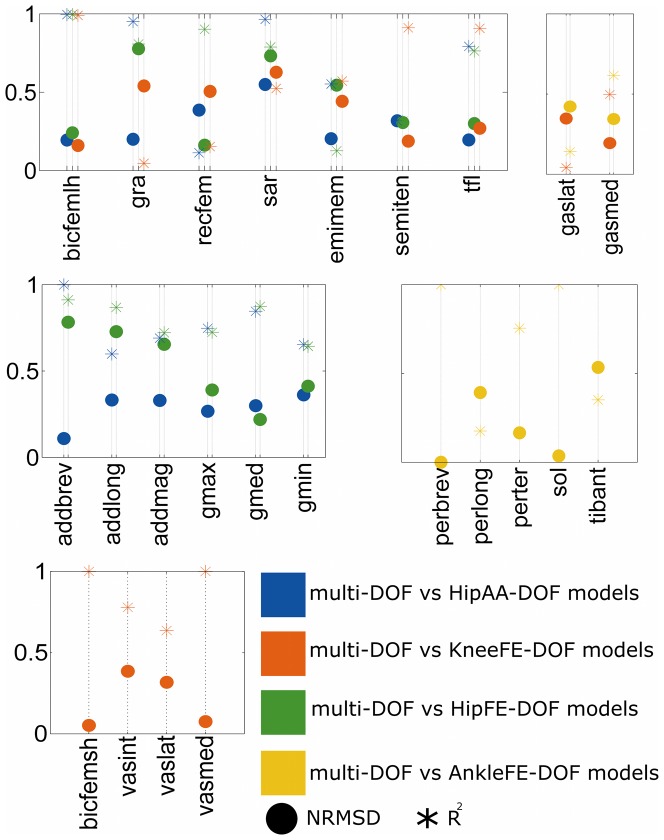
Third test results: Comparing MTU force estimates between single-DOF and multi-DOF models. Square of the Pearson product moment correlation coefficient (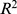) and normalized root mean squared deviation (NRMSD) between musculotendon unit (MTU) forces predicted by the multi-DOF model and by the single-DOF models custom-made to the four degrees of freedom (DOFs): hip adduction-abduction (HipAA), hip flexion-extension (HipFE), knee flexion-extension (KneeFE), and ankle flexion-extension (AnkleFE). MTUs names are defined as in Figure 2. Values for the addmag, gmin, gmax, and gmed have been reported as the average between the individual values associated to the three units each muscle is composed of (Figs. 2a, d, and e).

The third test (Figure 5) compared multi-DOF and single-DOF models 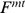
solutions for all MTUs revealing the multi-DOF model prediction strategy corresponded to a mixture of single-DOF model prediction strategies. The multi-DOF model solutions were similar both in shape and in magnitude to the single-DOF models solutions for a subset of biarticular hip-knee MTUs including tensor fasciae latae (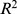
>0.77 and *NRMSD*<0.3 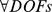), biceps femoris long head (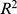
>0.98 and *NRMSD*<0.25 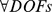), rectus femoris (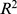 = 0.90 and *NRMSD* = 0.16 for the HipHFE-DOF), and semitendinosus (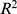 = 0.91 and *NRMSD* = 0.18 for the KneeFE-DOF), for a subset of uniarticular hip MTUs including gluteus maximus (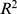
>0.73 and *NRMSD*<0.38 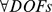), and gluteus medius (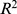
>0.85 and *NRMSD*<0.29 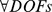), for a subset of uniarticular ankle MTUs including peroneus brevis (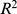 = 0.99 and *NRMSD* = 0.01), peroneus tertius (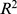 = 0.76 and *NRMSD* = 0.16), and soleus (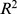 = 0.99 and *NRMSD* = 0.04), and for a subset of uniarticular knee MTUs including vastus medialis (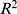 = 0.99 and *NRMSD*<0.08), and biceps femoris short head (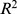 = 0.99 and *NRMSD* = 0.05).

The multi-DOF model solution for gracilis and adductor brevis were highly similar in shape to both the HipAA-DOF (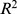 = 0.95, and 0.99 respectively) and HipFE-DOF (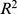 = 0.80, and 0.91 respectively) models solutions but similar in magnitude to only the HipAA-DOF model solution (*NRMSD* = 0.16, and 0.11 respectively). The multi-DOF model solution for the sartorius was similar in shape to the HipAA-DOF (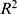 = 0.96) and to the HipFE-DOF (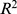 = 0.78) models solutions but different in magnitude with respect to all single-DOF models solutions (*NRMSD*>0.55 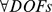). For the remaining MTUs, the multi-DOF and single-DOF models solutions were substantially different both in shape and in magnitude (Figure 5).

**Figure 6:**
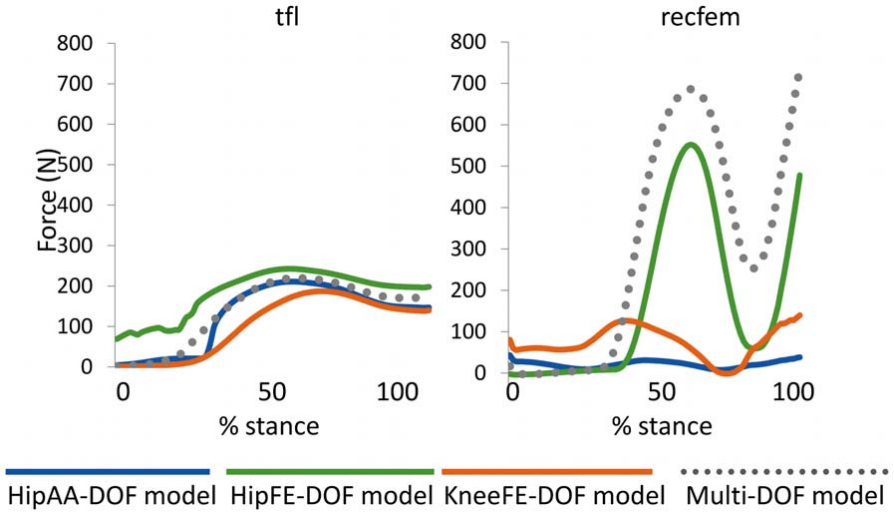
MTU force estimates between single-DOF and multi-DOF models. Ensemble average curves for the force predicted for the tensor fascia latae (tfl) and the rectus femoris (recfem) musculotendon units (MTUs) over 10 running trials. The reported data are from the stance phase with 0% being heel-strike and 100% toe-off events. MTU forces were predicted by the multi-DOF model and by the single-DOF models custom-made to the three degrees of freedom (DOFs) spanned by the two MTUs: hip adduction-abduction (HipAA), hip flexion-extension (HipFE), and knee flexion-extension (KneeFE).

A further comparison of results from the biarticular rectus femoris and tensor fasciae latae was conducted to better show the limitations associated to single-DOF 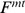 solutions and for demonstrating how the 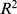 and the *NRMSD* coefficients relate to differences across single-DOF 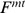 solutions (Figure 6). In this, the two MTUs were chosen as they represented two opposite conditions: 1) the rectus femoris, with single-DOF model solutions denoted by low shape similarity and by high magnitude differences, and 2) the tensor fasciae latae, with single-DOF model solutions denoted by a high shape similarity and by low magnitude differences. For the rectus femoris the KneeFE-DOF and HipAA-DOF models estimated a negligible contribution for this MTU to running, which were substantially different from the solution produced by the HipFE-DOF model that predicted a substantial contribution of this MTU. Figure 6 also depicts the multi-DOF model solution which was similar in shape and in magnitude to that estimated by the HipFE-DOF model (also see Figure 5). On the other hand, single-DOF estimates for the tensor fasciae latae were well correlated with each other's as well as with the corresponding multi-DOF estimate (Figures 5 and 6).

The fourth test revealed that the average calibration time for the multi-DOF model was 20 h and 35 min and the average execution time was 18.4±0.4 ms.

## Discussion

This study investigated the limitations associated to the currently available state of the art EMG-driven musculoskeletal models that constrain MTUs to satisfy joint moments with respect to a single DOF. It also aimed to show the importance of using a more comprehensive EMG driven model that accounts for multiple DOFs and for a larger set of MTUs. To this end, we developed a lower extremity musculoskeletal model that used EMG signals recorded from 16 muscle groups to predict the force developed by 34 MTUs. Furthermore, we developed a calibration procedure that allowed calibrating the multi-DOF model parameters so that the estimated MTU forces: 1) were consistent with experimental EMGs, 2) were the result of physiological MTU operation, and 3) satisfied joint moments about multiple DOFs over different contractile conditions associated to four motor tasks: WK, RN, CO, and SS. Results showed that the models calibrated with respect to different single-DOFs generated different MTU force solutions for the same input data and MTU set. On the other hand, our proposed multi-DOF model provided a unique MTU force solution that satisfied all DOFs and was therefore more generalizable because it was not specific to an individual DOF.

The first test showed that although the same EMG signals and joint kinematics were used to drive the four single-DOF models, they produced substantially different force predictions for all MTUs, implying multiple solutions to the MTU force prediction problem (Figure 3). This would lead one to the question about which single-DOF model to choose to analyze the MTU force contribution, especially for MTUs that span multiple joints. Furthermore, if the predictions across the MTUs that are shared by different single-DOF models do not match, then how would one combine different single-DOF solutions to predict MTU force and joint moment about multiple DOFs and joints? These are major limitations of using single-DOF models that were never pointed out in the literature and provided the main reason for the need of a multi-DOF model.

The second test results showed that the multi-DOF model could concurrently predict joint moments with similar accuracy to those from the four single-DOF models (Figure 4). For some DOF moments, in particular tasks, the multi-DOF model performed better than the equivalent single-DOF calibrated model. Therefore, the inclusion of multiple DOFs in the model structure did not influence the accuracy in joint moment prediction with respect to a particular DOF.

The third test demonstrated the ability of the multi-DOF model of using mixed single-DOF prediction strategies across MTUs (Figure 5). This allows accounting for the fact that different MTUs may not necessarily use a specific single-DOF prediction strategy but rather a combination of different single-DOF prediction strategies simultaneously. This suggests our proposed methodology can further generalize the MTU behavior than previously presented single-DOF models.

Furthermore, experimental results suggested that our proposed multi-DOF modeling methodology could produce EMG-driven MTU solutions that better reflect the actual activated MTUs than single-DOF models. In the case of the rectus femoris the multi-DOF model produced force estimates that better matched with previously obtained findings (Figure 6) [11], [34]. It is known that during running the rectus femoris is active to mostly generate hip flexion, especially in late stance and early swing [11], [34]. However, the KneeFE and HipAA-DOF models estimated a negligible contribution of this MTU that did not reflect its physiological behavior. The reason is that the KneeFE-DOF and HipAA-DOF models did not account for the rectus femoris ability of generating moment about the HipFE-DOF during running. On the other hand, the multi-DOF calibrated model predicted a force of this muscle that was consistent with its generally accepted role as it properly constrained the rectus femoris operation to both the knee and the hip joints. This provided confidence that our proposed methodology can properly constrain EMG-driven MTUs to satisfy multiple DOF dynamics to better reflect the way lower extremity muscles respond to the mechanical demand during movement.

Experimental results also showed that both the single-DOF and multi-DOF EMG-driven models could not produce joint moment estimates that exactly matched the experimental joint moments (Figure 4). This is in part related to two main limitations of surface EMG: 1) the inability to access EMG data from deeply located MTUs, and 2) difficulties in characterizing the EMG frequency bandwidth to best drive the musculoskeletal model [35]. First, surface EMG does not permit the activity of deeply located MTUs to be measured. In the current study this meant the EMG-dependent active forces generated by the illiacus and psoas MTUs could not be predicted. This resulted in the HipFE joint moment estimated during walking being smaller than the associated experimental moment during the hip-flexing phase of stance (i.e. 70%–100%) (Figure 4). The absence of the deep hip flexor EMG-dependent force contribution would also affect the prediction of hip flexion moments during the swing phase. Second, limitations in characterizing the appropriate EMG frequency bandwidth resulted in the EMG-driven model's inability to predict the high frequency components of the experimental joint moments (Figure 4). EMG linear envelopes are obtained by high-pass filtering, rectifying, and low-pass filtering the raw EMG signals using pre-defined cut-off frequencies. In the literature there has been a great debate about the most appropriate cut-off frequencies to be used in this process. Potvin and Brown [36] showed that using high-pass cut-off frequencies greater than 100 Hz resulted in significant and substantial improvements in MTU force estimates. Alternatively, Bobet and Norman [37] suggested that the high-pass filtered and rectified signal should then be low-pass filtered with a cut of frequency in the range of 1–3 Hz. Others suggested cut off frequencies in the range of 5–30 Hz for high-pass filtering and 3–10 Hz for low-pass filtering [8], [10], [12], [17]. This confusion across the literature is probably due to the use of time-invariant cut-off frequencies that fail at representing the continuous modulation of the frequency spectrum of the neural excitation. This is in fact a function of multiple variables, including the dynamics of the neural drive, the muscle contraction effort, and the dynamics of the task [9]. A potential approach to limit the above-mentioned problems is the use of hybrid EMG-driven/optimization-driven procedures in which the activity of MTUs that cannot be measured is predicted using an optimization-based approach. Furthermore, the experimentally measured EMG linear envelopes can be continuously adjusted in the time and frequency domains to account for tracking errors of the joint moment high-frequency components [38]. Alternatively, extracting motor unit action potentials using high-density EMG grids permits extracting richer information on the actual muscle neural excitation and on its continuous frequency bandwidth modulation. Motor unit action potentials directly reflect the discharge in the axons of the motor neurons innervating the motor unit and therefore represent a direct measurement of the neural drive to muscles. However, although high-density EMG represents an exciting solution to address current limitations in EMG-driven modeling, it is currently only feasible during isometric conditions [9].

The present results showed that the multi-DOF model could predict MTU forces and joint moments within the range of DOFs, tasks, and gait cycle phases (i.e. stance phase) on which the model was calibrated. However, how the model predicts (i.e. extrapolates) outside the range of these DOFs, tasks, and gait cycle phases requires an extensive and structured research, which was beyond the scope of this study. It could be that there are certain DOFs, trials, and gait cycle phases that provide better control over the final calibrated model than other calibration data, and that these data enable robust extrapolation across all manner of tasks, DOFs, and phases. This is important to be determined as the size of the calibration data set also affects the speed at which calibration can occur. Indeed, our proposed multi-DOF model relies upon an off-line calibration procedure that was time consuming. However, the fourth test results showed that the model open-loop operation could be performed in a time close to that of the muscles electromechanical delay (i.e. between 10 ms and 20 ms) [39]. Future work should also focus on the design of faster calibration algorithms. In this context, the use of muscle models that do not require an explicit integration of the MTU dynamics equations could considerably speed up the calibration process as it was shown in [15].

This work presented a study on one subject only and, therefore, may not be completely generalizable. However, the proposed EMG-driven musculoskeletal model was scaled and then calibrated to the actual subject to account for the subject-specific 1) anthropometry, 2) EMG-to-activation mapping, and 3) MTU intrinsic properties. First, with regards to the anthropometric scaling, our procedure linearly scaled the mass distribution and dimensions of every segment in the model as well as the MTU insertion, origin, and MTU-to-bone wrapping points according to the subject's anthropometry [26]. Second, the EMG-to-activation filtering coefficients have a day-to-day variation due to a number of factors such as the electrode placement and position, as well as the skin impedance. However, studies have shown that the re-calibration of these parameters can well account for different electrodes positioning by maintaining high joint moment prediction accuracy across testing sessions [11], [20]. Therefore, this validation did not need to be re-examined in the current study. Third, the non-linear scaling and then calibration procedure were used to determine the subject's MTU parameters including: tendon slack length, optimal fiber length, and maximal isometric force. This procedure used initial estimates obtained using the preferred method presented by Winby et al. [14], which were further calibrated to the individual subject using the non-linear optimization procedure as previously outlined. This procedure could in general account for subjects with varying muscular strength and operating ranges, [10], [11], [13], [14] thereby permitting potential investigations on people with musculoskeletal pathologies. Indeed, the calibration step was successfully used in people who suffered stroke [40], [41], and who had anterior cruciate ligament deficits [18]. Studies have also shown that, once the MTU tendon slack length, optimal fiber length, and maximal isometric force have been properly identified, their values do not change, at least over the short term [11], [20]. In this scenario, these parameters need to be identified only once for the specific subject [10], [11]. However, this assumption might not be valid when the subject's muscle function properties are altered as a result of dramatic changes in their exercise habits, disease status, or neurorehabilitation treatments. This scenario would require the MTU parameters to be re-calibrated. Nevertheless, despite the limited sample in the current study, the scaling and calibration procedures allow our methods to be applied across individuals without relying on the existence of specific anthropomorphic models, while accounting for the individual's muscle activation patterns across multiple DOFs. This represents an improvement in current state of the art methodologies were the recruited subjects were chosen to be of similar build of the anatomical model [11], [42]. However, a more general model validation across different pathologies will be the subject of future work.

Our proposed methodology predicted joint moments during the stance phase only. The main reason for this was that calibration included trials of running, as well as sidestepping and crossover cutting maneuvers. For these motor tasks the swing phase occurred partially, or totally, out of the motion capture volume. Therefore there was an incomplete swing phase data available for calibration across trials. The second, although much lesser reason, was that joint moments were estimated using inverse dynamics, which strongly relies on the magnitude of GRFs [26]. During the swing phase of locomotion, the GRFs are zero, which means the inverse dynamics calculations become highly sensitive to segmental inertial parameters that are difficult to measure *in vivo*. These include the segment mass, the location of the segment center of mass, and the mass moment of inertia [26], [43], which were only scaled linearly to the subject's size. Inverse dynamics measurements of joint moments during the swing phase may therefore not be reliable and we preferred to not use these for the model calibration and for the subsequent validation step. Future work will focus on 1) using better methods for extracting subject-specific segmental parameters (e,g using MRI), and 2) predicting joint kinematics rather than joint moments, using full forward dynamics models [44], or non-parametric methods such as Bayesian filtering [45]. This will allow extending the analyses presented in this study to the whole gait cycle thus increasing the applicability of our proposed methodology.

Future work is also needed to validate whether the use of experimentally derived subject-specific anatomy and MTU parameters from imaging techniques (i.e. MRI and ultrasound) [46] and the use of high-density electromyography [47] could increase the EMG-driven model reliability and decrease the influence of the off-line calibration that was observed to be substantially high in single-DOF models. However, improvements in current imaging techniques and high-density electromyography are still needed to allow applying these methodologies to the study of a large set of MTUs and individuals during dynamic movement [47].

The above-mentioned points refer to limitations that apply to any EMG-based musculoskeletal modeling methodology. In this context, it is important noting that the aim of our proposed work was not that of addressing all those limitations in one single study. Our aim was to demonstrate the clear disadvantages of single-DOF EMG-driven modeling and that the associated limitations could be addressed using our proposed multi-DOF modeling methodology. In this scenario, this work needed to compare the two methods under the same conditions including: subject sample, motor task sample, EMG signal processing procedure, and model calibration and scaling procedure.

In conclusion, this work proposed a novel EMG-driven musculoskeletal model of the human lower extremity and a calibration procedure that allowed identifying individual MTU parameters that could be used to predict a single MTU force solution, from experimentally measured EMGs, that was dynamically consistent with multiple DOF moments. Results showed that our proposed methodology could be applied to study dynamic movement and account for different muscle contractile conditions (i.e. different motor tasks). Our experimental results suggested that the multi-DOF model solutions better reflected the way MTUs respond to the mechanical demand resulting in a more accurate reflection of the actual muscle behavior than it was possible with previously proposed single-DOF models. This may help obtaining a deeper understanding of the neuromuscular dynamics during the human movement and better addressing the important scientific questions previously approached using single-DOF EMG-driven modeling. The proposed methodology may also have direct implications in neurorehabilitation technologies especially for the design of EMG-based human-machine interfaces for the control of powered orthoses and prostheses that imply the simultaneous actuation of multiple joints.
